# Analysis of clinical and neurological outcomes in patients with cauda equina syndrome caused by acute lumbar disc herniation: a retrospective-prospective study

**DOI:** 10.18632/oncotarget.20453

**Published:** 2017-08-24

**Authors:** Si-Dong Yang, Feng Zhang, Wen-Yuan Ding

**Affiliations:** ^1^ Department of Spinal Surgery, The Third Hospital of Hebei Medical University, Shijiazhuang, 050051, PR China; ^2^ Department of Rehabilitation Medicine, The Third Hospital of Hebei Medical University, Shijiazhuang, 050051, PR China; ^3^ Hebei Provincial Key Laboratory of Orthopaedic Biomechanics, Shijiazhuang, 050051, PR China

**Keywords:** cauda equina syndrome, lumbar disc herniation, spine, risk factor

## Abstract

**Objectives:**

In this research we analyzed the results of surgical treatment of cauda equina syndrome (CES) caused by acute lumbar disc herniation. We emphasize the early treatment for good neurological recovery.

**Methods:**

A retrospective-prospective, non randomized, clinical study was performed between Jan 2010 and Dec 2014. We retrospectively collected medical records of 18 patients who suffered from CES due to acute lumbar disc herniation and followed up them regularly. Visual analogue scale (VAS) score, lumbar JOA score (29 points), RR (recovery rate) and Oswestry disability index (ODI) questionnaire were used to evaluate clinical outcomes.

**Results:**

All patients were followed up for at least two years. Lumbar disc herniation is located at L2-3 level in 2 cases, L3-4 level in 2 cases, L4-5 level in 9 cases, L5-S1 level in 5 cases. VAS score is 6±2.5 preoperatively and 1.5±1.0 postoperatively at last follow-up (P<0.001). JOA score is 5±3.5 preoperatively, while it is 20±7 postoperatively at last follow-up (P<0.001). RR ≥ 50% was found in 12 cases. ODI is 75%±25% preoperatively, while it becomes 28%±16% postoperatively at last follow-up (P<0.001). It also shows that advanced age (≥45 years) may act as a risk factor for poor RR(<50%), while early operation (duration before surgery, <48 h) proves to be a protective factor.

**Conclusions:**

Early operations are mandatory and closely relevant to final outcomes for CES patients. However, elder patients are more likely to have poor clinical effect after surgery.

## INTRODUCTION

Cauda equina syndrome (CES) is a syndrome of symptoms and signs not all of which need to be present to make diagnosis; there is no agreed definition of CES [1]. As regards to CES, there are five characteristic features including bilateral neurogenic sciatica, reduced perineal sensation, altered bladder function ultimately to painless urinary retention, loss of anal tone and sexual dysfunction [2]. Two clinical categories of CES are recognized on the basis of whether it is complete or partial [3]. In complete CES, there is complete urinary retention and severe bowel dysfunction. In incomplete CES, there is reduced urinary sensation and partial loss of bowel function. The incomplete CES patient has objective evidence of CES but retains voluntary control of micturition although there may be other disturbances of micturition such as urgency, poor stream, hesitancy and/or reduced bladder or urethral sensation [4]. The symptoms of CES vary differently depending on the location of the injury in cauda equina. The most essential reason is the compression of spinal nerve roots [5].

Acute CES is an uncommon but significant neurologic presentation due to a variety of underlying diseases. Anatomical compression of nerve roots, usually by a lumbar disc herniation is a common cause in the general population, while inflammatory, neoplastic, and ischemic causes have also been recognized [6]. Nucleus pulposus herniation at L3-S1 levels has been always observed clinically [5]. A persistent neurologic impairment, although generally improved after the onset of the syndrome, is a condition that strongly affects patients’ quality of life and restricts their social activities [7].

Herein, the aim of this study is to investigate clinical outcome related to patients with CES caused by acute lumbar disc herniation. In addition, risk factors for poor recovery are explored in this study.

## RESULTS

**Figure 1:**
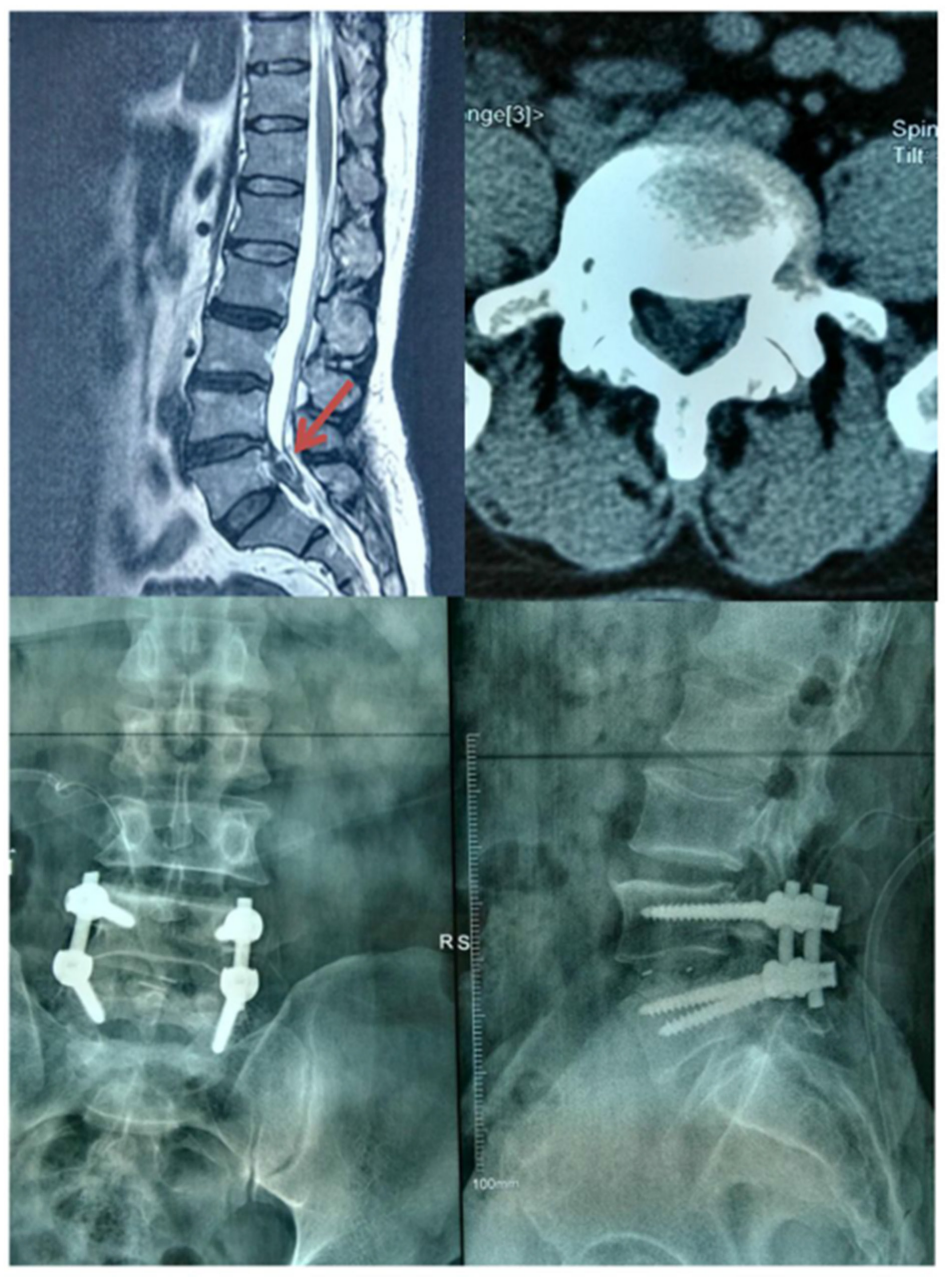
The prolapsed disc was removed from spinal canal after an emergency operation performed And an X-ray radiograph was taken postoperatively.

Of the 18 patients, 11 underwent emergency operations within 48 h. Three patients cannot tolerate an emergency operation due to poor body status. The other 4 patients had lost the best operation opportunities within 48 h before they were admitted to our hospital. All patients were followed up for at least two years, with a median of 36 months. Lumbar disc herniation is located at L2-3 level in 2 cases, L3-4 level in 2 cases, L4-5 level in 9 cases, L5-S1 level in 5 cases. As shown in Figure 1, the patient suffered from acute L4-5 disc herniation resulting in CES and then underwent decompressive laminectomy and disectomy with pedicle screw fixation and intervertebral fusion.

As shown in Table 1, VAS score is 6±2.5 preoperatively and 1.5±1.0 postoperatively at last follow-up, which is statistically significant (P<0.001). However, it is of no significance between 1-year follow-up group and last follow-up group.

**Table 1:** Comparison of VAS score with preoperative data by LSD-t test

Group	Preoperative	3 months	6 months	1 yr	last follow-up
Score	6±2.5	4.0±2.2	2.5±1.2	2.0±1.4	1.5±1.0
t	-	2.29	5.35	5.92	7.09
P-value	-	0.028	<0.001	<0.001	<0.001

VAS, Visual Analogue Scale.

As shown in Table 2, JOA score is 5±3.5 preoperatively, while it is 20±7 postoperatively at last follow-up with statistical difference (P<0.001). RR ≥ 50% was found in 12 cases. As shown in Table 3, comparison of RR regarding patient age reveals that younger patients recover better than elder patients do (P=0.043). In addition, comparison of RR regarding duration before surgery reveals that early surgical treatment is better for the recovery of patients (P=0.017), as shown in Table 4.

**Table 2:** Comparison of JOA score with preoperative data by LSD-t test

Group	Preoperative	3 months	6 months	1 yr	last follow-up
Score	5±3.5	13.5±5	15±7	17±8	20±7
t	-	5.91	5.42	5.83	8.13
P-value	-	<0.001	<0.001	<0.001	<0.001

JOA, Japanese Orthopaedic Association.

**Table 3:** Comparison of RR regarding patient age

Group	n (age≥45)	n (age<45)
RR≥50%	3	9
RR<50%	5	1
Fisher's Exact Test		P =0.043

**Table 4:** Comparison of RR regarding duration before surgery

Group	n (≥48h)	n (<48h)
RR≥50%	2	10
RR<50%	5	1
Fisher's Exact Test		P =0.017

As shown in Table 5, ODI is 75%±25% preoperatively, while it becomes 28%±16% postoperatively at last follow-up (P<0.001). Although ODI at last follow-up became less than that at 1-year follow-up, no significance was found between them (P>0.05).

**Table 5:** Comparison of ODI with preoperative data by LSD-t test

Group	Preoperative	3 months	6 months	1 yr	last follow-up
Score(%)	75±25	55±20	50±16	35±20	28±16
t	-	2.65	3.64	5.30	6.72
P-value	-	0.012	<0.001	<0.001	<0.001

ODI, Oswestry disability index.

As shown in Table 6, it showed that lower-limb muscle strength recovered well after the operations by comparing the postoperative lower-limb muscle strength with the preoperative status according to MRC classification (P<0.001).

**Table 6:** Comparison between preoperative and postoperative lower-limb muscle strength

Group	grade 0	grade I	grade II	grade III	grade IV	grade V
Pre	0	1	3	8	6	0
Post	0	0	1	3	4	10

Pre, preoperation; Post, postoperation at last follow-up.

P<0.001, comparing postoperative lower-limb muscle strength to the preoperative status.

## DISCUSSION

CES is a rare but serious condition, defined as “a spectrum of low back pain, uni or bilateral sciatica, saddle anesthesia and motor weakness in the lower extremities with variable rectal and urinary symptoms” [8]. Its incidence is 1 in 33,000 to 100,000, and it occurs with 2% of all lumbar disc herniations [9]. Bowel and sexual disturbances can be part of clinical presentation at diagnosis but most frequently they become clear later as a consequence of the potentially irreversible neurologic damage of the nerve roots of the cauda equina. Acute CES is an uncommon but significant neurologic presentation due to a variety of underlying diseases. Anatomical compression of nerve roots, usually by a lumbar disc herniation is a common cause in the general population, while inflammatory, neoplastic, and ischemic causes have also been recognized [6].

To our knowledge, what constitutes CES, how it should be subclassified and how urgently to image and operate on patients with CES are all matters of debate. The conclusions from a recent study [2] suggest an emergency surgery for such a patient with bilateral radiculopathy, a large central disc prolapse, and cauda equina nerve root compression. The patient in this case report underwent emergency operation with the surgeon's decision based on his urgent symptoms and pathological signs. Clearly, the management for the patients in our department appear consistent to the recommended guideline by that study.

In our study, VAS score, JOA score and ODI were investigated and found that most patients recovered to a good level after at least two-year follow-up. It could be the reason that most patients in this study are young and early operations were performed within 48 hours of CES onset, which was revealed by comparison of RR regarding duration before surgery showing that early surgical treatment is better for the neurological recovery. And it is consistent with a previous study [10], which reported that, commonly, patients with complete sensory recovery were operated within 48 hours of symptom onset. Therefore, in most patients early surgery was associated with better outcome. However, some patients may have lost the best opportunities of an emergency surgery after CES appeared. Clinically, the reasons vary differently for the patients who did not take an urgent operation within 48 hours. The body status was too poor for some patients, some with high blood pressure or severe diabetes mellitus, and some others with heart diseases. Thus, they cannot tolerate an emergency operation. In addition, some other patients went to some smaller hospitals near their families and received conservative treatment there for several days after the syptoms appeared. So an early emergency operation was impossible for those patients.

As we know, the reasons leading to acute disc herniation are various. Of them, spinal massage and spinal manipulation have been reported much. Up to now, spinal massage has already been repeatedly reported regarding serious adverse events it leads to [11–18]. Massage may result in acute spinal subdural hematoma [12], cervical hematomyelia [13], subcutaneous hematoma [14], spinal accessory neuropathy [16], lumbar epidural hematoma [17]; but most are CES [15, 17, 18]. Data from those reports indicate that massage may be associated with mild and transient adverse events, but sometimes it may be indeed associated with some serious complications leading to permanent disability, or even death.

Tamburrelli et al [15] reported a 42-year-old patient who complained a rapid onset of saddle hypoparesthesia and urine retention only a few hours after spinal massage performed for L5-S1 herniated disc. Obviously, it was an urgent case needing an emergency surgery. A close pathogenetic relationship with spinal massage was then confirmed by the MRIs performed before and after the massage. The patient finally referred an incomplete recovery at 1 year follow-up after undergoing an emergency operation. Solheim et al [17] reported the first case of a lumbar epidural hematoma at L3 level after chiropractic manipulation which resulted in partial CES with lower extremity paresis and urinary retention. During the follow-up period, the patient's motor deficits improved, but the bladder dysfunction remained after surgical evacuation of the hematoma performed through laminectomy of L3 and L4.

The exact risk of injury caused by spinal manipulation is still unknown. The serious incidence of adverse events was estimated ranging from 1.46 case series in 10 million manipulations to 5 strokes in 100,000 manipulations, and a death rate of 2.68 in 10 million manipulations has also been reported [19, 20]. Some studies [21, 22] on spinal manipulation revealed that 30% to 55% patients reported a minor adverse event. Most of all were local discomfort (53% to 60%), radiating discomfort (10% to 23%), headache (10% to 12%), tiredness (11%), or nausea; dizziness, hot skin, or “other” reactions are uncommonly reported (<5% of reactions). Most reactions were mild, moderate, or transient.

## MATERIALS AND METHODS

### Ethics statement

This study has been approved by Ethics Committee of the Third Hospital of Hebei Medical University. The approval number is K2017-02-01.

### Patients

Medical records of 18 patients with CES caused by acute lumbar disc herniation were collected between Jan 2010 and Dec 2014, including 14 males and 4 females, with an average age of 47 years old (ranging from 25 to 71 years). All cases were admitted into our hospital with following symptoms, back pain, leg pain with numbness, weakness of lower extremities, accompanied by urine retention or incontinence, and/or constipation or incontinence of faeces, and/or sexual dysfunction. All underwent decompressive laminectomy and disectomy except 4 cases performed with pedicle screw fixation and intervertebral fusion to avoid further instability.

### Postoperative follow-ups and outcome assessment

The clinical follow-up observation was carried out by outpatient review. The patients were regularly followed up rigorously by independent evaluators at 3, 6, 12 months after operation. After then, the patients were followed up once every year. All patients were followed up at least two years. The last follow-up result was used as post-operation evaluation index. Clinical effectiveness was evaluated by visual analogue scale (VAS) score, Japanese Orthopaedic Association (JOA) score (29 points) and Oswestry Disability Index (ODI) score. Preoperative and postoperative lower-limb muscle strength was assessed according to MRC (the UK Medical Research Council) classification. The recovery rate (RR) of JOA score was calculated according to the following formula: RR = (postoperative scores – preoperative scores)/(29 - preoperative scores) ^*^100%. Based on the preoperative, postoperative and last follow-up scores, the RR was calculated. According to RR, clinical effect was divided into four grades. RR ≥ 50%, good; 10% ≤ RR <50%, moderate; 0 % ≤ RR <10%, poor; RR <0%, deteriorated. However, we just have 18 patients identified in this study since CES are not so common as other neuro-spinal diseases. The small sample size may limit the detailed comparisons. Thus, we only performed comparisons regarding RR ≥ 50% and RR<50%.

### Statistical analysis

Statistical analyses were performed using SPSS for Windows, version 18.0 (SPSS Inc., USA). Data are presented as Mean ± SD (standard deviation) for measurement data. For count data, it was presented as percentage, and chi-square test or Fisher exact test was used for data analysis. VAS/JOA/ODI score preoperatively and that at follow-ups after operation were compared using repeated measurement ANOVA (analysis of variance), accompanied by LSD-t test for pairwise comparison. A p-value less than 0.05 was considered as statistically significant.

## CONCLUSIONS

Our results have confirmed that early operations are mandatory and closely relevant to final outcomes for CES patients. However, elder patients are more likely to achieve poor clinical outcomes after surgery.

## Abbreviations

CES: cauda equina syndrome
VAS: visual analogue scale
RR: recovery rate
ODI: Oswestry disability index
JOA: Japanese Orthopaedic Association
SD: standard deviation
ANOVA: analysis of variance
MRC: the UK Medical Research Council
